# Shape of the Pulmonary Doppler Sonography Blood Flow Profile of the Congo Grey Parrot (*Psittacus erithacus*) and the Influence of Heart Disease

**DOI:** 10.3390/vetsci12050468

**Published:** 2025-05-14

**Authors:** Carolin Girard, Norbert Kummerfeld, Michael Pees, Michael Fehr, Marko Legler

**Affiliations:** Department of Small Mammal, Reptile and Avian Medicine and Surgery, University of Veterinary Medicine Hannover, Foundation, Bünteweg 9, 30559 Hanover, Germany

**Keywords:** sonography, systolic blood flow, acceleration time, deceleration time, acceleration phase, deceleration phase, parrots

## Abstract

In avian medicine, sonographic examination is an important diagnostic tool for heart diseases. Little is known about the diagnostic relevance of the Doppler blood flow profiles of parrots. In the present study, sonographic examinations of Congo grey parrots with atherosclerosis were evaluated retrospectively to gain more knowledge about their pulmonary and aortic systolic blood flow. The shapes of their Doppler sonographic blood flow profiles were quantified by the determination of the acceleration and deceleration phases. The investigations showed the differences between the aortic flow profile, with fast rising velocities, and the pulmonary flow profile, which has a round shape, in grey parrots. Diseased parrots with ultrasonographic signs of a right heart failure, such as an enlarged right ventricle and/or insufficiencies of the right atrioventricular and/or pulmonary valve, showed a significantly shorter acceleration and longer deceleration phase than parrots without heart failure or with sonographic signs of left heart failure only. The correlation of the shape of the pulmonary Doppler blood flow profile with the diameter of the left atrium, the systolic and diastolic diameter of the right ventricle, the fractional shortening of the left and right ventricle, and the mean aortic and pulmonary blood flow illustrates the importance of these sonographic parameters in the investigation of the function of the right ventricle in heart disease. Our examination showed that the shape of the pulmonary Doppler blood flow profile of Congo grey parrots provides important information about the pressure load on the right ventricle in heart diseases, which is comparable to that in small animal and human medicine.

## Simple Summary

Cardiovascular diseases are common in avian patients in captivity, and especially in parrots. In these cases, sonographic examination is an important diagnostic tool for investigating heart failure. In the present study, the shape of the systolic pulsed-wave Doppler blood flow profiles of Congo grey parrots with a presumed heart disease due to atherosclerosis were examined retrospectively and correlated with other sonographic findings associated with left and especially right heart failure. The results of the present study illustrate the influence of heart disease, and particularly right heart failure, on the shape of the pulmonary artery blood flow profile. Thus, our investigations show how helpful the evaluation of the blood flow profile of the pulmonary artery can be in the assessment of heart disease and the prevailing pressure conditions in the heart in bird patients such as the Congo grey parrot.

## 1. Introduction

Cardiovascular diseases are well known in avian medicine [1,2,3]. The most common cause of heart failure in birds in captivity, especially parrots, is atherosclerosis [1,2,3]. Moreover, out of all parrots, Congo grey parrots are the most susceptible to developing atherosclerosis [1,2,3]. This vascular disease can cause heart failure due to the reduced elasticity of the blood vessels and a tightening of the lumen of arteries, which lead to an increase in the afterload, secondary cardiomyopathies, and valvular insufficiencies [2,4,5]. Other groups of diseases that can influence heart function are, for example, primary cardiomyopathies, myocarditis and diseases of the heart valves which result in insufficiencies, systemic hypertension, pulmonary hypertension, or septal defects [1,2,6]. These diseases are accompanied by clinical signs that differ in severity depending on the stage of heart failure [2]. Common signs indicating heart failure in bird patients are dyspnea, exercise intolerance, and, especially in the case of arteriosclerosis, cramping seizures or cramped feet, often seen as falling off the perch [1,2,6]. However, it has to be kept in mind that clinical signs may be nonspecific and can be accompanied by signs of other diseases, such as pulmonary or liver diseases, which may disguise the clinical picture [2].

Diagnostic possibilities in avian cardiology are limited compared to those in small animal or human medicine [1,2]. Radiology is a well-established technique in avian medicine and can help in diagnosing heart disease by showing pathological changes in the heart silhouette and vascular disease through an increase in the X-ray density of the affected vessels [1,2,7]. Some studies show that computer tomography (CT) and positron emission tomography (PET) scans can also be used to diagnose atherosclerosis in birds through the spatial display of blood vessels, with PET scans being more sensitive to smaller lesions in the vessels [8,9,10]. A lack of reference values for many avian species and the availability of the medical equipment can make the usage of these diagnostic tools difficult. Cardiac ultrasonographic examination provides a more precise insight into heart function, although its practical implementation depends on the species-specific and individual shape of the air sacs and sternum of the birds. Those factors, along with the size of the avian patient, can complicate the ultrasonographic display of the heart, though especially improved ultrasound devices enable the use of Doppler echocardiography in avian cardiology [1,2,11,12,13,14,15]. Measuring the blood flow in the heart during diastole and systole can contribute to the assessment of heart disease, even though reference values are missing for a lot of avian species [16,17,18,19].

Previous studies on the systolic blood flow of racing pigeons have shown that the blood flow of the aorta (AO) and pulmonary artery (PA) differ significantly from each other [14,20], as already reported in small animal and human medicine [18,21,22,23]. The differences in the Doppler sonographic flow profiles of the AO and PA are assumed to be a result of the different pressure situations in the left and right heart [21,22,23]. The lower pressure system of the right heart and pulmonary circulation produces a pulmonary artery Doppler flow profile (PAFP) in which the acceleration phase (AP) is similar in duration to the deceleration phase (DP), resulting in a rounded shape [18]. In contrast, the Doppler flow profile of the aorta (AOFP) features a short AP and a longer DP, reflecting the higher pressure system of the systemic circulation and left heart [18,24].

Studies in both small animals and human indicate that diseases causing pulmonary hypertension significantly affect the shape of the PAFP, as the increased pressure in the pulmonary circulation results in a PAFP with a short AP, similar to that of the AOFP [23,25,26,27,28,29,30].

The purpose of this pilot study was to retrospectively evaluate the shape of the PAFP and AOFP in Doppler sonographic examinations conducted in Congo grey parrots (*Psittacus erithacus*) with an assumed heart disease due to atherosclerosis. The relationship between these profiles and the diastolic and systolic blood flow velocities of the heart, as well as selected diameters of the left and right ventricle, are to be investigated to uncover further information about the effects of heart disease on the shape of the pulmonary Doppler blood flow profile of Congo grey parrots.

## 2. Materials and Methods

We retrospectively evaluated Doppler sonographic images from patients that were examined in the department following a standardized protocol between 2010 to 2020.

### 2.1. Examined Congo Grey Parrots

**Figure 1:**
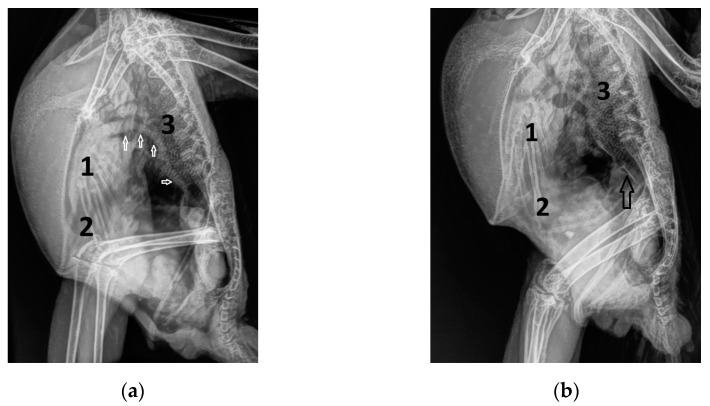

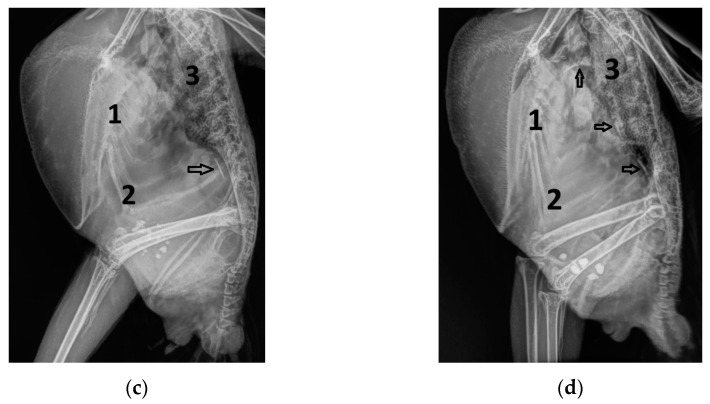
Laterolateral radiographs of Congo grey parrots with atherosclerosis: (**a**) with a hyperdense aorta without calcifications (arrow) and a normal heart silhouette (GP from group 2); (**b**) with a hyperdense aorta with calcifications (arrow) and a normal heart silhouette (GP from group 1); (**c**,**d**) with hyperdense aorta with varying degrees of calcifications (arrow) and signs of heart failure, such as an enlarged heart silhouette, lung edema, and an enlarged liver silhouette due to effusion into the hepatoperitoneal cavity (GP from group 3); 1: heart silhouette; 2: liver; 3: lungs.

In this retrospective study, Doppler and B-Mode ultrasonographic data from 118 Congo grey parrots (GPs) were examined. The length of the sternum of the GPs, as measured on the laterolateral radiographs, was 71.7 mm ± 4.1 standard deviation (SD) (Xmin: 60 mm; Xmax: 82 mm). The GPs were presented to the clinic with varying clinical signs indicating heart failure, mainly poor performance, dyspnea, and convulsion, or for a preventive medical examination. In all cases, the GP showed calcifications or hyperdensity of its large vessels in the radiographs, an indication of atherosclerosis (Figure 1) with (*n* = 94) or without clinical signs of heart disease (*n* = 24). The heart index (the measurement of the width of the cardiac silhouette in relation to the width of the thorax) was used to determine whether there was an enlarged heart silhouette. The clinical signs of the examined parrots were classified from 1 to 5 (1: no clinical signs; 2: minimal apathy; 3: minimal apathy, short convulsion, stress intolerance, and stress-induced dyspnea; 4: moderate apathy, moderate dyspnea in relation to pulmonary edema, clear circulatory failure; 5: highly impaired general health, high degree dyspnea in relation to pulmonary edema, and high degree circulatory failure). Cases with clinical signs that were solely due to calcium deficiency were excluded. Follow-up sonographic examinations were included as cases in this study if there were changes in heart size, valve function, and/or clinical signs. Therefore, a total of 118 individual GPs led to 149 sonographic cases that were categorized independently and included in this study.

All cases of diseased GPs were classified into three groups using the diameter of their ventricles and valvular insufficiencies. The ventricle was classified as enlarged if it was larger in systole and/or diastole than the simple standard deviation of the reference values published by Pees et al. [1]. Valvular insufficiencies were diagnosed by coloured Doppler sonography. Cases were classified as group 1 if there was no cardiac enlargement and no valvular insufficiencies were detected by sonography and as group 2 if there was an enlargement of the left ventricle and insufficiencies in the aortic valve (AOV) and/or the left atrioventricular valve (AV). The cases that were classified as group 3 showed an enlargement of the right ventricle and/or insufficiencies of the pulmonic valve (PAV) and/or of the right AV. If, in addition to the affected right heart, the left heart was also involved to a degree that matched the specifications of group 2, the case was classified as group 3 as well.

In the sonographic examinations, attention was also paid to pericardial effusion, cavity effusion, sonographic abnormal liver tissue (liver disease), and the movement of the septum in the direction of the left ventricle in systole.

### 2.2. Doppler Sonographic Examination

All echocardiographic images were acquired by using a 10 MHz phased-array transducer (GE 10S-RS Probe; B Mode 4.5–11.5 MHz; GE VINGMED ULTRASOUND A/S, Horten, Norway) in conjunction with a digital ultrasound system (Vivid 7 Dimension BT08; GE VINGMED ULTRASOUND A/S, Horten, Norway) in the clinic’s routine diagnostics. Only in a few parrots were the sonographic images combined with an electrocardiogram (ECG; GE VINGMED ULTRASOUND A/S, Horten, Norway). The ECG was taken on the left and right patagium and the left flank fold using clamps without teeth, in accordance with Einthoven [31]. During the sonographic assessment, the parrots were awake and secured in a semi-upright position. This enabled the ventro-median approach to performing cardiac ultrasonography. The longitudinal horizontal view (“Four and Five Chamber view”) of the heart was used to visualize the anatomy of the heart, the left and right ventricle, and the atria7, depending on systole and diastole (B-Mode) [11,32,33]. One way to display the blood flow in the pulmonary artery is to examine the right outflow tract using a horizontal view, starting with a four-chamber view with a visible AO and correcting the probe under colour flow Doppler imaging [14].

The 2D images were overlaid with colour flow Doppler information from the ultrasound system to verify the function of the heart valves. The field with colour flow Doppler information was set to be as small as possible to guarantee frame rates above 80 frames per second. The pulse repetition frequency (PRF) value was adapted individually to the anticipated blood flow velocities of each patient, between 1 and 11.76 kHz, to reduce aliasing. Depending on the PRF, a wall filter of 6.54 to 19.63 cm per second was used. The sample volume setting was 0.4 mm. Flow towards the transducer was colour-coded in red and flow away from the transducer was colour-coded in blue. The sonographic examinations were stored on the ultrasound system and evaluated in slow motion and as single images.

Pulsed-wave (PW) Doppler sonography with a sample volume of 1.5 mm was chosen to examine the blood flow velocities in the area of the heart valves. A setting for the wall filter of 3.4 cm/s was chosen to eliminate motion artefacts from cardiac structures. The angle correction function was utilized for the measurements.

Continuous-wave (CW) Doppler can measure faster velocities and was used to characterize severe insufficiencies.

### 2.3. Measurments of the Heart Diameter and of Doppler Blood Flow Profiles

Various sonographic parameters were measured, and their abbreviations are shown in Table 1. The measurements were taken from stored examinations using the GE device software (Vivid 7 Dimension BT08, V.7.3.0) either on the ultrasound devices or with the software EchoPAC^TM^ Version 204 (GE VINGMED ULTRASOUND A/S, Horten, Norway). The dimensions of the heart were measured from B-Mode images over three heart cycles, including end-systolic and end-diastolic diameters. The atria were measured at their largest extent (width and height) and the elliptic area was calculated. The mean was used for further evaluations. The systolic (Vs) and diastolic (Vd) ventricular diameters were used to calculate the fractional shortening (Vd − Vs/Vd × 100) of the left and right ventricle.

The blood flow velocities during systole and diastole, depending on the heart frequencies, were evaluated in metres per second (m/s) over six consecutive heart cycles. The blood flow time (ejection time; ET) was calculated in milliseconds (ms) from the beginning to the termination of the flow. The curve function of the ultrasound system was used to measure the systolic mean velocities. The mean was used for further evaluations.

The shape of the blood flow profiles of the aorta and pulmonary artery was quantified by measuring the acceleration time (AT) from the onset of ejection to the peak flow velocity, as well as the deceleration time (DT) from the peak flow velocity to the end of the blood flow, in ms, depending on the heart rate. The percentages of AT and DT relative to the ET were calculated to quantify the AP and DP, thereby characterizing the flow profiles. The AP and DP were utilized for further correlation analyses. All measurements were taken over six consecutive individual heart beats, and their mean values were used for subsequent evaluations.

### 2.4. Statistical Analysis

Statistical analyses were conducted using SPSS^®^ Statistics 29 (IBM, Armonk, NY, USA). The mean, median, standard deviation (SD), and range (Xmin to Xmax), as well as 25% and 75% quartiles, were calculated for the parameters of the systolic and diastolic Doppler flow profiles. The Kolmogorov–Smirnov test indicated that some parameters were not normally distributed. Consequently, the differences among the various parameters of the blood flow profiles of the aorta and pulmonary artery were assessed using the Mann–Whitney U test. Spearman’s rank correlation coefficient (r) was employed to illustrate the influence of the heart rate and to demonstrate correlations between systolic and diastolic blood flow velocities and heart diameters. A significance level of *p* ≤ 0.05 was established. A correlation coefficient r of 0.1 < 0.3 was regarded as a low correlation, 0.3 < 0.5 as a moderate correlation, and 0.5 < 0.7 as a strong correlation.

## 3. Results

As this study was retrospective, not all sonographic parameters were recorded in every examination of the clinically affected GPs, and, therefore, they could not always be investigated. In total, the PAFP could be assessed in 114 and the AOFP in 137 cases of the 149 echocardiographic examinations of 118 GPs.

**Figure 2:**
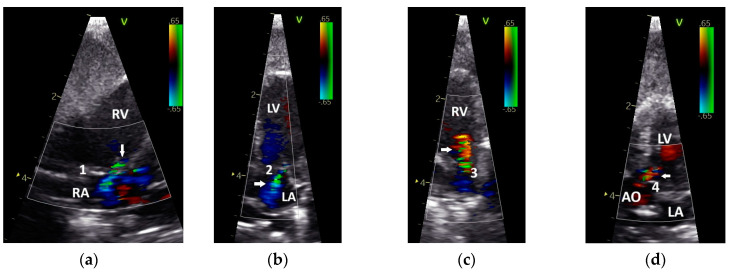
Insufficiencies of the right (**a**) and left (**b**) atrioventricular valve and the pulmonary (**c**) and aortic valve (**d**) of Congo grey parrots, visualized by colour flow Doppler echocardiography. Arrow: marked insufficiency jet; RV: right ventricle; LV: left ventricle; RA: right atrium; LA: left atrium; AO: aorta; 1: right atrioventricular valve; 2: left atrioventricular valve; 3: pulmonary valve; 4: aortic valve. The colour scale on the right side of the image is calibrated in m s^−1^.

**Figure 3:**
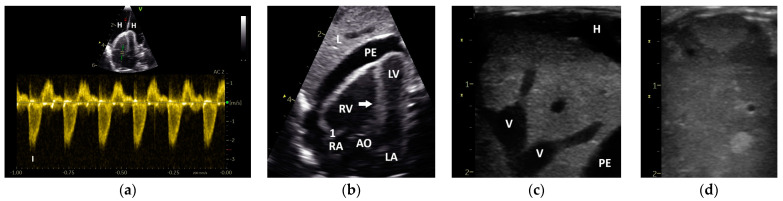
Sonographic findings of Congo grey parrots: (**a**) Insufficiency of the right atrioventricular valve (AV_right_) due to an enlarged right ventricle, with PW Doppler sonographic diastolic blood flow profile of the AV_right_ with a systolic insufficiency jet (I) with a very short acceleration phase; H: hepatoperitoneal cavity effusion. (**b**) End-diastolic movement of the septum in the direction of the left ventricle (arrow), as seen with B-Mode echocardiography; PE: pericardial effusion; RV: right ventricle; LV: left ventricle; RA: right atrium; LA: left atrium; AO: aorta; L: liver; 1: AV_right_. (**c**) B-Mode sonography of a congested liver associated with right heart failure, with compacted liver tissue and highly congested liver veins (V); H: hepatoperitoneal cavity effusion; PE: pericardial effusion. (**d**) B-Mode echocardiography of a liver with nodular liver tissue associated with the shape of the pulmonary blood flow profile, which had a short acceleration phase (outlier group 1).

Based on their sonographic findings, the GPs were divided into three groups: group 1: cases without sonographic evidence of heart disease (26 cases from 24 GPs); group 2: cases with left heart failure (60 cases from 46 GPs); and group 3: cases with right or with left and right heart failure (63 cases from 53 GPs). The applied classification criteria resulted in clear differences between the groups (Table 2 and Table 3). For example, in group 2, only insufficiencies of the AOV (*n* = 10) and the AV_left_ (*n* = 25) were seen, while in group 3 insufficiencies of the AOV (*n* = 4), the AV_left_ (*n*= 16), the PAV (*n* = 2), and the AV_right_ (*n* = 18) could be detected (Table 2; Figure 2). The investigations showed dilatations of the heart ventricles in 34 cases from group 2 and in 63 cases from group 3 (LV: *n* = 4; RV: *n* = 27; RV and LV: *n* = 22; Table 3). In group 3, in some cases with right heart failure, a clear end-diastolic movement of the septum towards the left ventricle (*n* = 7; Figure 3) could be determined. Furthermore, also in group 3, more clinical signs of congestion in the form of hepatoperitoneal cavity effusion (*n* = 10) and pericardial effusion (*n* = 9) could be detected (Table 2; Figure 3).

Fractional shortening (FS) was evaluated as a parameter of the ventricular function. There were significant differences between the cases from different groups. A decrease in the FS of the left ventricle could be detected especially from group 1 to groups 2 and 3 (*p* ≤ 0.05; Mann–Whitney U test). A significant decrease in the FS of the right ventricle could be detected especially from groups 1 and 2 to group 3, which illustrates the occurrence of right ventricular failure in group 3 (Table 4).

The Doppler sonographic results for each group are shown in Table 5, Table 6 and Table 7. Their heart rates during the measurement have also been included, since they are important for interpreting the findings.

The width of the cardiac silhouette measured from the ventrodorsal radiographs (“heart score”) was significantly different between the three groups (*p* < 0.02; Mann–Whitney U test; Table 8).

In general, the clinical signs displayed by the groups did not differ significantly. A significant difference was only found between groups 2 and 3, with group 3 showing the more severe clinical signs (*p* = 0.01; Mann–Whitney U test).

The focus of the investigations in the present study was the shape of the systolic Doppler blood flow profiles of the aorta and pulmonary artery. These results are presented in the following subsections.

### 3.1. Shape of PAFP and AOFP of Grey Parrots

**Figure 4:**
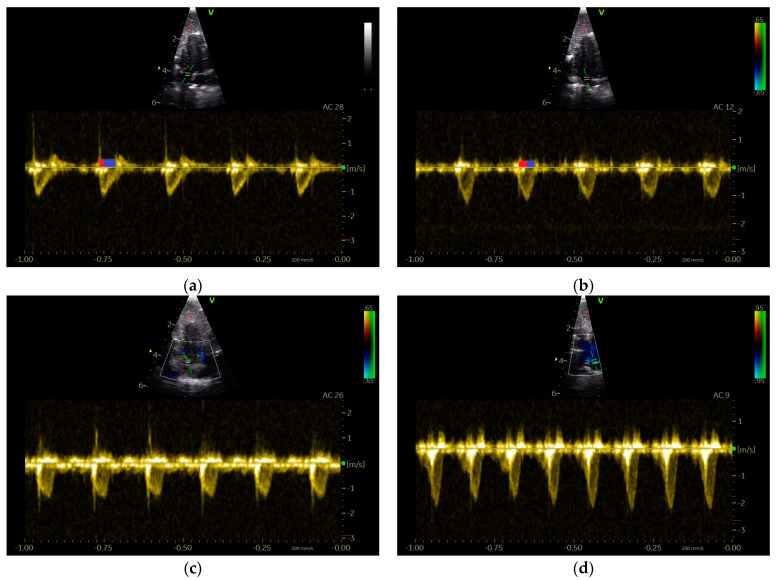
PW Doppler sonographic blood flow profiles of the aorta (**a**) and pulmonary arteries (**b**–**d**) of Congo grey parrots without sonographic evidence of heart disease (GPs from group 1). The acceleration phase of the blood flow is visible in red and the deceleration phase in blue in (**a**,**b**). A sample gate is placed in the area of the aortic (**a**) or pulmonary valve (**b**–**d**); AC: angle correction. The colour scale on the right side of the images is calibrated in m s^−1^.

Our examinations of the systolic Doppler flow profiles of the GPs revealed a significant difference between the shape of their PAFP and AOFP. The percentage of the AT in the total ET of the AOFP and PAFP, as a reflection of the blood flow profile’s shape, indicated that the acceleration phase of the blood flow of the PA was significantly longer, while the deceleration phase of the PAFP was significantly shorter compared to the AOFP (*p* < 0.001; Mann–Whitney U test) in the examined GPs (Figure 4). The PAFP exhibited a rounded shape with an AP that was slightly shorter than the DP (*p* < 0.05; Mann–Whitney U test) in the examined GPs that did not display any sonographic evidence of heart disease (group 1; Table 6). The AOFP was defined by a short AP and a longer DP (*p* = 0.005; Mann–Whitney U test; group 1; Table 5; Figure 4).

**Figure 5:**
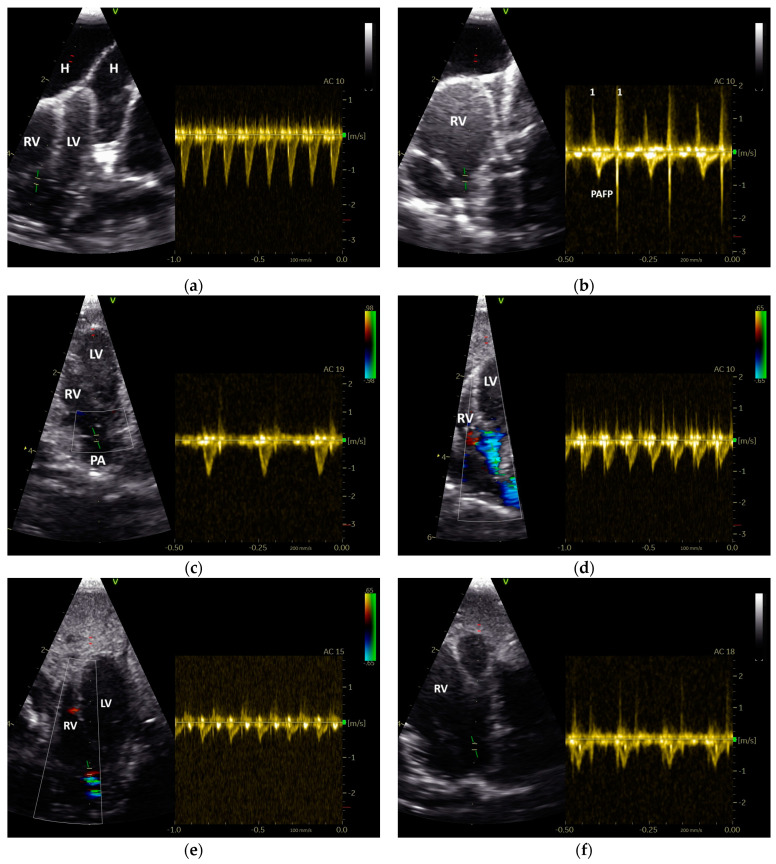
PW Doppler sonographic blood flow profiles of the pulmonary artery of Congo grey parrots with left and right heart failure ((**a**–**f**); GPs from group 3). In all presented cases, the abnormally short acceleration phase (AP) of the pulmonary blood flow profile (PAFP) is visible; RV: right ventricle; LV: left ventricle; PA: pulmonary artery; H: hepatoperitoneal cavity effusion; 1: Doppler signal of the pulmonary valve, with a sample gate placed in the area of the pulmonary valve; AC: angle correction. The colour scale on the right side of the images is calibrated in m s^−1^.

However, in GPs with left heart (group 2) and/or right heart failure (group 3), we found a shortening of the AP of their PAFP (Table 5, Table 6 and Table 7). The PAFP showed a short AP and thus the PAFP looks similar to that of the aorta, especially in advanced cases (Figure 5).

**Figure 6:**
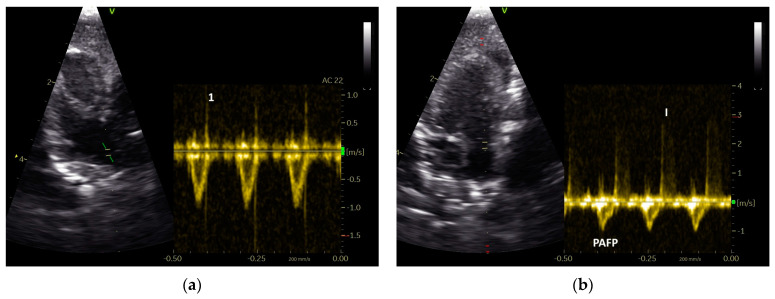
PW Doppler sonographic blood flow profiles of the pulmonary artery of a Congo grey parrot that developed right heart failure over the course of one year: (**a**) a symmetric blood flow profile of the pulmonary artery (PAFP), without signs of heart failure, and (**b**) the shape of the PAFP a year later, with a short acceleration phase seen in conjunction with the development of right heart failure displayed as right ventricular dilatation and an insufficiency of the pulmonary valve. I: insufficiency jet; 1: Doppler signal of the pulmonary valve, with a sample gate placed in the area of the pulmonary valve; AC: angle correction.

In one case from the group of GPs without sonographic evidence of heart disease (group 1), a PAFP with a very short AP, causing it to look similar to the AOFP, which was associated with a nodular liver disease (Figure 3), was found. In some GPs, a change in the shape of the PAFP was recognizable and connected to a worsening of the apparent disease, which led to a new grouping. For example, a clear change in the shape of the PAFP in connection with the development of a pulmonary insufficiency (Figure 2) and right heart dilatation (right heart failure) over one year is presented in Figure 6.

In our examinations, the aortic ET was not significantly different to the systolic flow time of the PA in the diseased GPs (*p* > 0.05; Mann–Whitney U test).

### 3.2. The Influence of Heart Failure on the Shape of the PAFP and AOFP

**Figure 7:**
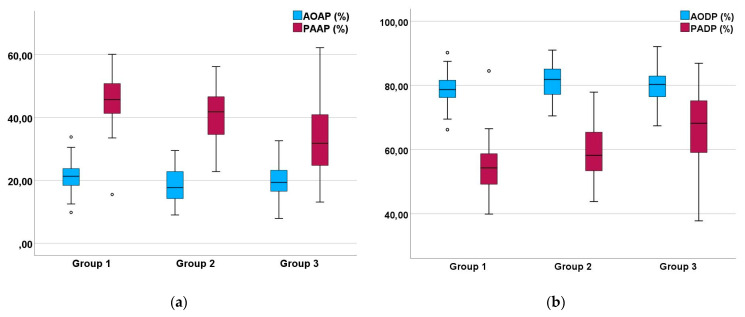
Comparison of the PW Doppler-derived acceleration phase (**a**) and deceleration phase (**b**) of the blood flow of the pulmonary artery and aorta of Congo grey parrots depending on the classification of their heart failure: group 1, cases without sonographic evidence of heart disease; group 2, cases with left heart failure; and group 3, cases with right or with left and right heart failure; The box plot shows the median (horizontal line in the centre), the 25th percentile (bottom box border), and the 75th percentile (top box border). The upper and lower ends of the whiskers show the maximum and minimum values. The single data points are outliers.

The investigations of the Doppler sonographic images of the diseased GPs revealed that the PAFPs were significantly different between the three groups (PAAP of group 1 to 2: *p* ≤ 0.05; group 1 to 3: *p* < 0.001; group 2 to 3: *p* = 0.004; Mann–Whitney U test). The longest PAAP was detected in cases without sonographic evidence of heart disease (group 1). Cases with an increasing involvement of the right heart in heart failure showed a significantly shorter PAAP and a longer PADP (group 1 to 2: *p* ≤ 0.05; group 1 to 3: *p* < 0.001; group 2 to 3: *p* = 0.009; Mann–Whitney U test). This could also be seen in group 2, with only cases with sonographic evidence of left heart failure showing a significantly shorter PAAP than cases without heart disease (group 1). These results are illustrated graphically in Figure 7. In contrast to the PAFP, the AOFP did not significantly vary between the different groups of diseased GPs (*p* > 0.05; Mann–Whitney U test; Figure 7).

A systolic septal movement to the left could be seen in seven cases from group 3. In these cases, the PAAP (mean 31.8% ± SD 7.0) was shorter than the mean value of PAAP for the other cases in this group, but not significantly (*p* > 0.05; Mann–Whitney U test).

### 3.3. Influence of the Heart Rate on the Pulmonary and Aortic Blood Flow Profiles of the GPs

The heart rate of the examined diseased GPs had a significant negative low-to-moderate influence on the ET and the AT of the systolic pulmonary and aortic blood flow, as shown in Table 9 (r = −0.2 to −0.40; *p* ≤ 0.05). The DT of the blood flow of the aorta was also significantly but lowly negatively influenced (r = −0.28; *p* < 0.001).

In contrast to the influence on the different time parameters of the blood flow, the shape of the blood flow of the PA and AO was not significantly influenced by the heart rate in the examined GPs (AP and DP of PA and AO: r = −0.002 to −0.07; *p* ≥ 0.46).

### 3.4. Correlation of Diastolic and Systolic Blood Flow Velocities with the Shape of the Pulmonary and Aortic Blood Flow Profiles in the Examined Diseased Grey Parrots

Various correlations between the blood flow velocities and the systolic blood flow patterns were identified in our study (Table 9). However, the shape of the PAFP was significantly correlated with the systolic blood flow velocities of the PA and AO. A longer AP and shorter DP were significantly by lowly correlated with higher peak blood flow velocities (PAAP: r = 0.15; *p* = 0.02; PADP: r = −0.14; *p* = 0.004) and mean blood flow velocities (PAAP: r = 0.20; *p* = 0.002; PADP: r = −0.19; *p* = 0.02) of the PA and higher mean blood flow velocities of the AO (PAAP: r = 0.18; *p* = 0.002; PADP: r = −0.19; *p* = 0.02). The peak blood flow velocities of the AO were not significantly lowly correlated with the shape of the PAFP (PAAP: r = 0.21; *p* = 0.08; PADP: r = −0.21; *p* = 0.08). The correlation between the shape of the PAFP and diastolic ventricular blood flow was not significant. But a negative, non-significant, low correlation was seen with EA_left_ (PAAP: r = −0.12; *p* = 0.08; PADP: r = 0.11; *p* = 0.09) and EA_right_ (PAAP: r = −0.12; *p* = 0.07; PADP: r = 0.12; *p* = 0.09).

The shape of the blood flow profile of the AO was significantly lowly correlated with the aortic mean blood flow velocities (AOAP: r = −0.19; *p* = 0.03; AODP: r = 0.19; *p* = 0.03), the peak blood flow (AOAP: r = −0.21; *p* = 0.03; AODP: r = 0.19; *p* = 0.05), and the mean blood flow velocities of the PA (AOAP: r = −0.23; *p* = 0.02; AODP: r = 0.20; *p* = 0.04). Additionally, it showed a low-to-moderate correlation with the diastolic blood flow of the left ventricle (AOAP: r = −0.31; *p* < 0.001; AODP: r = 0.27; *p* = 0.002). The peak blood flow velocities of the aorta were not correlated with the shape of the AOFP (AOAP: r = −0.03; *p* = 0.75; AODP: r = 0.03; *p* = 0.75).

A significant correlation between blood flow velocities and the ET of the PA and AO was seen only with the diastolic left blood flow velocities, EA_left_, and the PAET (low correlation: r = 0.13; *p* = 0.05)

### 3.5. Correlation of the Pulmonary and Aortic Blood Flow Profiles with Different Heart Diameters

The correlations of the pulmonary and aortic blood flow profiles with different heart diameters are summarized in Table 10.

The shape of the PAFP was lowly correlated with the diameter of the left atrium (PAAP: r = −0.15; *p* = 0.05; PADP: r = 0.15; *p* = 0.05) and the diameter of the right ventricle (RVd: PAAP: r = −0.21; *p* = 0.02; PADP: r = 0.21; *p* = 0.004; RVs: PAAP: r = −0.27; *p* < 0.001; PADP: r = 0.26; *p* < 0.001). Furthermore, a non-significant low correlation with the diameter of the RA was seen (PAAP: r = −0.29; *p* = 0.07).

The shape of the AOFP was significantly correlated with only the systolic left ventricular diameter (AOAP: r = −0.20; *p* = 0.03; AODP: r = 0.23; *p* = 0.02). Non-significant low correlations of the AOFP with the LVd (AOAP: r = −0.16; *p* = 0.07; AODP: r = 0.16; *p* = 0.07) the LA (AOAP: r = −0.18; *p* = 0.07; AODP: r = 0.18; *p* = 0.07) were observed and a non-significant moderate correlation with the RA was also seen (AOAP: r = 0.31; *p* = 0.09; AODP: r = −0.34; *p* = 0.06).

As a functional parameter, the fractional shortening was also significantly lowly correlated with the PAFP and the AOFP. Higher values of the FS of the left and right ventricle were significantly correlated with a longer PAAP (FSLV: r = 0.15; *p* = 0.02; FSRV: r = 0.23; *p* < 0.001) and a shorter PADP (FSLV: r = 0.23; *p* = 0.003; FSRV: r = −0.21; *p* = 0.002). The shape of the AOFP was only significantly correlated with the FS of the right ventricle (AOAP: r = −0.23; *p* = 0.01; AODP: r = 0.20; *p* = 0.03).

The systolic ejection times of PA and AO were also significantly correlated with some heart diameters. The PAET was significantly lowly positively correlated with the diameter of the LV (r = 0.13; *p* = 0.05) and RV (RVs: r = 0.18; *p* = 0.01; RVd: r = 0.21; *p* = 0.02) in the systole phase and significantly moderately correlated with the RA (r = 0.38; *p* = 0.02). The PAET was significantly negative and lowly correlated with the FS of the LV (r = −0.19; *p* = 0.003) and RV (r = −0.14; *p* = 0.03). The ET of the systolic blood flow of the AO was significantly positively correlated with the LA (r = 0.29; *p* = 0.004) and non-significantly lowly positively correlated with the LV in the systole phase (r = 0.16; *p* = 0.05) and RV in the diastole phase (r = 0.15; *p* = 0.09) and negatively correlated with the FS of the LV (r = −0.15; *p* = 0.09).

## 4. Discussion

The aim of this retrospective study was to evaluate the shape of the PAFP and AOFP of Congo grey parrots (*Psittacus erithacus*), as detected by PW Doppler sonography. To the best of our knowledge, this study is the first on the shape of the systolic blood flow in relation to heart disease in birds [1,2,13,20].

Our investigations showed that Doppler sonography is an important technique for visualizing and examining the blood flow in the AO and PA in bird patients, which have naturally high heart frequencies, such as grey parrots [1,2,11,14]. There is little research on the interpretation of systolic blood flow in relation to heart disease in birds to date [2,19]. In particular, information on the sonographic examination of the blood flow of the PA of GPs is completely missing [14,16,24]. In the cases examined in the present study, the blood flow in the AO was investigated more frequently, emphasizing that the visualization of the PA in birds is more difficult, as also described in the literature [1,14]. However, the echocardiographic examinations of the GPs evaluated in the present study followed the same techniques and device settings as previously described for racing pigeons [14,20,24]. In particular, the right outflow tract was visualized using a horizontal view, starting from a five-chamber view with the AO visible and correcting the ultrasound probe, which was in some cases under the control of colour Doppler imaging, as previously described in pigeons [14,20,24].

The evaluation of the Doppler sonographic examinations of the systolic blood flow of GPs without sonographic evidence of heart disease, group 1 in the present study, revealed that there are clear differences in the blood flow of the AO and the PA in GPs (Figure 7). The pulmonary artery Doppler flow profile showed a rounded shape, with an acceleration phase comparable to the deceleration phase. In contrast to the PAFP, the Doppler flow profile of the aorta was characterized by a very short AP and a long DP. These conditions, which we found in GPs, are the same as those described in pigeons and as those already studied in small animals and human medicine [14,18,20]. Blood flow profiles are an expression of the pressure ratios in the heart and circulation [18]. More specifically, the different pressure situations in the lower pressure system of the right heart and the pulmonary circulation compared to the higher pressure system of the left heart and the body circulation can be assumed to be the explanation behind the different shapes of the PAFP and AOFP, comparable to in small animal medicine [2,19,34,35]. Precise investigations of the pressure conditions in the heart of GPs are completely lacking, especially in diseased individuals, whereas it is a standard examination technique in human medicine [36,37]. However, there are subtle differences between the PAFPs of GPs and racing pigeons. The AP (45.3% ± 9.9) of the PAFP of awake GPs without a sonographically detectable heart failure, group 1 in the present study, is significantly shorter than the AP of racing pigeons (53.3 ± 7.0) [20]. In this context, it should be kept in mind that all parrots examined in the present study were diseased animals, even if they did not show sonographic evidence of heart failure. This is particularly visible in the classification of their clinical signs, which did not differ between the defined groups. In addition, all animals were sonographically examined because they showed radiographic signs of a vascular disease, atherosclerosis. Furthermore, it seems possible that diseases, even if they are not primary cardiovascular diseases, can have an influence on the pressure of the vascular system and thus affect the flow profiles of the heart, such as pulmonary mycosis, tumours, or liver diseases. The differences between these bird species could also be explained by the naturally higher blood pressures and principally increased stress levels in grey parrots compared to racing pigeons. This is also reflected in the different heart rates of GPs (395.5 beats/min ± 96.8) and pigeons (207.5 beats/min ± 46.0) [17], which were detected under the same sonographic conditions. Moreover, in studies of the direct arterial blood pressure measurement of different bird species under anesthesia, pigeons showed a lower blood pressure than parrots [2]. A direct influence of the heart rate on the shape of the blood flow profiles of the PA and AO of diseased GPs could not be proven in the present study.

In the group of the GPs without sonographic evidence of heart disease, there was no overlap in the percentages of the AP and DP of the PAFP and the AOFP (Figure 7). However, the parrots examined were sick birds; therefore, our study does not provide normal values for Congo grey parrots. These values will have to be established in the future.

In contrast to this, in the groups of GPs with sonographic evidence of heart disease (group 2 and 3), we were able to find a significant shortening of the AP of the PAFP and thus an approximation of the shape of the PAFP to that of the AOFP, which was most pronounced in cases with right heart failure. In human and small animal medicine, a short AP of the PAFP indicates an increased pressure in the right heart due to volume overload, a higher pressure in the pulmonary circulation, or a higher vascular resistance in the lungs [25,26,27,29,36,37,38,39]. Therefore, our findings provide important insights into the pathophysiology of heart disease in avian medicine. Our results showed that the AP of the PAFP is also significantly reduced in cases of left heart failure without sonographic evidence of right heart failure (Figure 7). These results illustrate the influence of left heart failure on the pulmonary circulation, and therefore on the afterload of the right heart (causing an increased afterload), in parrots, especially in cases of congestive heart failure due to atherosclerosis [2]. In these cases, heart disease starts with the onset of left heart failure and is followed by right heart failure [2]. The results of our study visualize the pressures that could be involved in the pathogenesis of right heart failure in this context in grey parrots, as also studied in human medicine [28,36]. The assumed pressures become even clearer in cases with right heart failure with insufficiencies of the right AV and right ventricular dilatation. These cases, group 3 in our study (Figure 7), had the significantly shortest AP values in their PAFPs. In some cases, the pressure load on the right heart might not only be visible through the shortened AP values of the PAFP but also in other sonographic findings, such as an end-diastolic movement of the septum in the direction of the left ventricle or signs of congestion in the liver. The shortest mean AP values of the PAFP were determined in cases with an end-diastolic septal movement in the direction of the left ventricle. In small animal medicine, such a septal movement also indicates a high-grade increase in pressure or right-sided volume overload [18,40,41]. In human medicine, the shape of the DP of the PAFP can also give an indication of the pressure load in the heart [25,29]. Flow profiles with mid-systolic notching and a flow pattern with a reduction in signal volume indicate a high pressure load on the right heart [25,29]. This might also be examined in avian patients in the future, as the images in Figure 5 show signs that could indicate notching.

Our results show that the shape of the flow profile cannot be used to diagnose a cardiac disease, but that a disease can be better characterized and clinical signs explained by assessing the pressure conditions with the help of the PAFP. An important indication for the assessment of the PAFP in human medicine is the diagnosis of pulmonary hypertension, a disease that also plays a major role in GPs for various reasons [2]. Pulmonary hypertension is a common condition, which may occur, for example, as a consequence of vascular, chronic left heart or lung disease, as described in human medicine and assumed in avian medicine [1,36,37].

One of our cases uniquely illustrates the connection between liver disease (Figure 3) and pulmonary hypertension, which was evident in a shortened AP of the PAFP, without sonographic evidence of heart disease, as seen in a GP from group 1 (outliner of Figure 7). In avian medicine, this correlation has not previously been found in the literature [2]. However, there are descriptions from human medicine of cardiopulmonary disease also occurring in the presence of advanced liver disease [42].

Unfortunately, pathological and histological examinations were only available in very few cases in the present study; thus, statements about the definite cause of the illnesses and heart disease in the examined GPs could only be made in a few cases. However, all examined parrots were suspected of having an underlying atherosclerotic disease, though whether this vascular disease led to cardiac insufficiency in all cases remains speculative. Therefore, we were only able to make a very rough classification of these cases into three groups, which could have been refined through more pathological examinations to gather more specific information. This should be done in other studies in the future. Perhaps this will help to specify the influence of disease on the shape of the blood flow profile further and to improve the clinical use of this technique. The classification of the cases into three groups based on their sonographically visible ventricular dilatation was also difficult in the present study. One concern was that some dilatations would be classified as normal due to the different body sizes of the GPs, with the use of a double standard deviation of the mean ventricular diameter of healthy GPs [1]. Thus, we decided to use the simple standard deviation as a limiting value in the present study. The increase in heart size between the groups is shown by the significant increase in the heart index from group 1 to 3, independent of the body size.

According to our investigations, the shape of the PAFP can be seen as a functional parameter for the assessment of pressure conditions connected to heart disease, which is comparable to human and small animal medicine [25,26,27,28,29,36,37,38,39]. In contrast, such significance could not be determined for the AOFP in our investigations. The strictly regulated high pressure system of the systemic circulation and the left heart probably allows only a slight change in the shape of the AOFP in diseased animals in connection with heart disease, which probably only becomes visible immediately before imminent death [18].

In avian medicine, there are various sonographic parameters that are used to diagnose heart disease [1,2,19], so one of the aims of this investigation was to correlate the shape of the PAFP with these parameters. For the right heart in particular, some interesting correlations were found that describe the relationships between various cardiac parameters and possible pressure conditions in the heart of parrots. The effect of the left heart’s function on the pulmonary circulation is particularly evident from the negative correlation of the diameter of the left atrium with the AP of the PAFP [18]. An enlarged left atrium is associated with increased pressure in the left heart or an increased volume load due to an insufficiency of the left AV [18]. This insufficiency was also diagnosed in half of the cases in group 2 in the present study. Thus, the diameter of the left atrium should be considered in the evaluation of the right heart in avian patients, as described in small animal medicine [18]. This correlation also confirms the assumption that a minor enlargement of the left ventricle or left AV insufficiency alone, without atrial dilatation, is less significant than when seen in connection with an atrial enlargement in bird patients [1,2].

The positive correlation of the FS of the left and right ventricle with the AP of the PAFP in diseased GPs reflects that a rather round symmetric shape of the PAFP is seen with good cardiac function in balanced pressure conditions. FS is influenced by preload, afterload, and contractility [1,2,18]. The correlation of the FS of the right ventricle with the shape of the PAFP illustrates that this parameter can be valuable for assessing the function of the right ventricle, even if it is more variable than FS of the left ventricle [1]. Thus, the FS of the right ventricle should be determined in the echocardiographic examination of the avian heart [1,2]. The negative correlation of the AP of the PAFP with the size of the right ventricle in systole and diastole illustrates that the dilatation of the right heart might be associated with an increased pressure load on the right heart due to an increase in afterload, which acts as a driving force, as assumed in the literature [1,2,18].

Correlations with Doppler-based blood flow velocities could also be determined in our investigations. High mean and peak flow velocities of the PA and the mean flow velocities of the AO within the range of the determined velocities were assumed to be caused by a low pressure in the pulmonary circulation, recognizable by the longer AP of the PAFP. There was no correlation with the peak flow velocities of the AO. This could mean that the aortic peak blood flow velocities are more variable than the mean blood flow velocities of the aorta. These correlations indicate that in the future, more attention should be paid to mean blood flow velocities in the sonographic evaluation of cardiac diseases in avian patients. The correlation of the PAFP with diastolic blood flow velocities was not significant. However, a negative correlation of the blood flow velocities with the AP of the PAFP was observed at the level of the AV. Thus, increasing blood flow velocities in this region may also indicate increasing pressures in the heart and should be examined in birds with heart disease [1,2,18]. However, in the present study, the E and A waves of the diastolic blood flow were fused. A wave velocities are especially influenced by heart rates, as previously described in pigeons [15]. Thus, the influence of various physiological factors, such as heart rate, can have a negative impact on the correlation between the PAFP and the diastolic blood flow [15,18].

Insufficiency jets were not considered in our investigations. The examination of this pathological blood flow can also provide important indications of pathological pressure conditions in the heart in small animal and human medicine and should also be investigated further in avian patients [15]. One case of a grey parrot with an insufficiency of its right atrioventricular valve in connection with right ventricular dilatation illustrates the possible use of an insufficiency jet to assess the pressure conditions in the heart of avian patients. The Doppler sonographic examination in this case revealed a profile for the insufficiency jet with a short acceleration phase, demonstrating the increased pressure in the right ventricle in the same way the short AP of the PAFP does (Figure 4).

Studying the shape of the systolic blood flow profiles, particularly the PAFP, helped to reveal important relationships between cardiac morphology and blood flow, and it provided insights into the driving forces behind cardiovascular disease in avian patients. Further studies must show whether the results seen in Congo grey parrots can be applied to other bird species and whether the Doppler sonographic examination of the PAFP can be utilized to diagnose specific cardiac diseases in birds.

## 5. Conclusions

This retrospective analysis of the shape of systolic Doppler blood flow profiles revealed significant differences between the AOFP and the PAFP in Congo grey parrots. Their AOFP is defined by a short AP and a long DP, whereas their PAFP exhibits a round shape with an AP that is comparable to the DP. Additionally, no effect of the heart rate on the shape of the blood flow profiles was observed.

The examination of the PAFP of grey parrots suffering from atherosclerosis provided insights into heart failure in birds. A significant decrease in the AP of the PAFP was found in parrots with sonographic findings of left heart failure, and even more so in parrots with sonographic signs of right heart failure, and can be assumed to be signal of the increasing pressure in the right heart and pulmonary circulation.

The correlation of the AP of the PAFP with other sonographic parameters, such as the dimension of the left atrium, the systolic and diastolic diameter of the right ventricle, the FS of the left and right ventricle, and the mean aortic or pulmonary blood flow velocities illustrates the importance of these parameters for assessing the functioning of the right ventricle during heart disease.

## Figures and Tables

**Table 1:** Abbreviations of Doppler sonographic parameters used in this publication.

Abbreviation	Unit	Doppler Sonographic Parameter
AO	-	Aorta
AOV	-	Aortic valve
PA	-	Pulmonary artery
PAV	-	Pulmonary valve
AV	-	Atrioventricular valve
AT	ms	Acceleration time
DT	ms	Deceleration time
ET	ms	Ejection time
AP	%	Percentage of AT on ET
DP	%	Percentage of DT on ET
PAAT	ms	AT of the blood flow of the PA
PADT	ms	DT of the blood flow of the PA
PAAP (%)	%	AP of the blood flow of the PA
PADP (%)	%	DP of the blood flow of the PA
PAET (ms)	ms	ET of the blood flow of the PA
PAV_max_ (m/s)	m/s	Peak flow velocities of the PA
E_RV_V_max_ (m/s)	m/s	Peak E wave velocities of the right heart
A_RV_V_max_ (m/s)	m/s	Peak A wave velocities of the right heart
E to A ratio _right_	-	E to A wave velocity ratio of the right heart
HR_PA_ (beats/min)	beats/min	Heart rate during measurements of the PA
AOAT (ms)	ms	AT of the blood flow of the AO
AODT (ms)	ms	DT of the blood flow of the AO
AOAP (%)	%	AP of the blood flow of the AO
AODP (%)	%	DP of the blood flow of the AO
AOET (ms)	ms	ET of the blood flow of the AO
AOV_max_ (m/s)	m/s	Peak flow velocities of the AO
E_LV_V_max_ (m/s)	m/s	Peak E wave velocities of the left heart
A_LV_V_max_ (m/s)	m/s	Peak A wave velocities of the left heart
E to A ratio _left_	-	E to A wave velocity ratio of the left heart
HR_AO_ (beats/min)	beats/min	Heart rate during measurements of the AO
LVd	cm	Left ventricle dimension end-diastolic
LVs	cm	Left ventricle dimension end-systolic
RVd	cm	Right ventricle dimension end-diastolic
RVs	cm	Right ventricle dimension end-systolic
LA	cm^2^	Left atrium area (B-Mode)
RA	cm^2^	Right atrium area (B-Mode)
FS_LV_	%	Fractional shortening LV
FS_RV_	%	Fractional shortening RV

**Table 2:** The incidence of valvular insufficiencies, end-diastolic movement of the septum in the direction of the left ventricle (SM), sonographically diagnosed liver disease (LD), hepatoperitoneal cavity effusion (H), and pericardial effusion (PE) in the examined Congo grey parrots from group 1, cases without sonographic evidence of heart disease; group 2, cases with left heart failure; and group 3, cases with right or with right and left heart failure.

	Insufficiencies				SM	LD	H	PE
	AOV	PAV	AV_left_	AV_right_				
Group 1	0/23 ^1^	0/20	0/23	0/20	0/26	1/26	0/26	0/26
Group 2	10/58	0/46	25/58	0/46	0/60	7/60	4/60	0/60
Group 3	4/56	2/48	16/56	18/50	7/63	15/63	10/63	9/63

^1^ Number of findings detected in each case/number of cases examined.

**Table 3:** The incidence of ventricular enlargement in the examined Congo grey parrots from group 1, cases without sonographic evidence of heart disease; group 2, cases with left heart failure; and group 3, cases with right or with right and left heart failure.

	LV	RV	LV and RV
Group 1	0/23 ^1^	0/20	0/20
Group 2	34/58	0/46	0/46
Group 3	4/61	27/50	22/50

^1^ Number of findings detected in each case/number of cases examined.

**Table 4:** The fractional shortening (FS) of the left and right ventricle of the examined Congo grey parrots from group 1, cases without sonographic evidence of heart disease; group 2, cases with left heart failure; and group 3, cases with right or with left and right heart failure.

	FS	Mean ± SD ^1^	Xmin–Xmax	Median	25% Percentile	75% Percentile
Group 1	FS_LV_ (%)	30.1 ± 8.8	14.0–47.8	30.7	23.2	36.4
	FS_RV_ (%)	55.4 ± 12.3	32.9–76.7	55.3	46.0	64.5
Group 2	FS_LV_ (%)	22.2 ± 9.1	2.6–48.1	22.2	16.3	27.3
	FS_RV_ (%)	55.8 ± 15.8	30.2–100.0	53.1	44.1	69.2
Group 3	FS_LV_ (%)	22.4 ± 10.8	2.6–56.9	23.0	14.6	29.5
	FS_RV_ (%)	38.5 ± 18.2	10.1–79.4	36.1	24.2	52.2

^1^ SD: standard deviation.

**Table 5:** Doppler sonographic parameters of the examined Congo grey parrots (GPs) without sonographic evidence of heart disease (group 1); 26 cases were created from the 24 GPs examined.

Parameter	Mean ± SD ^1^	Xmin–Xmax	Median	25% Percentile	75% Percentile
PAAT (ms)	23.6 ± 8.3	8.8–47.5	24.3	18.0	28.4
PADT (ms)	27.8 ± 7.0	19.3–48.2	27.7	21.4	31.8
PAAP (%)	45.3 ± 9.9	15.5–60.1	45.7	41.2	51.8
PADP (%)	54.7 ± 9.9	39.9–84.5	54.3	48.3	58.9
PAET (ms)	53.5 ± 10.9	37.0–85.3	52.6	46.3	59.7
PAV_max_ (m/s)	1.57 ± 0.80	0.65–3.69	1.31	1.10	1.82
PAV_mean max_ (m/s)	1.12 ± 0.61	0.48–2.83	0.93	0.78	1.24
HR_PA_ (beats/min)	395.5 ± 96.8	240.0–600.0	390.0	465.0	480.0
EA_RV_V_max_ (m/s)	0.61 ± 0.13	0.41–0.84	0.60	0.51	0.74
HR_EARV_ (beats/min)	396.7 ± 101.6	240.0–600.0	360.0	322.5	480.0
AOAT (ms)	10.7 ± 2.6	6.0–18.0	10.5	9.0	12.6
AODT (ms)	41.7 ± 8.3	28.8–55.5	40.3	35.2	50.2
AOAP (%)	21.2 ± 5.5	9.8–33.8	21.3	18.2	24.5
AODP (%)	78.9 ± 5.5	66.2–90.2	78.7	75.5	81.8
AOET (ms)	56.6 ± 8.7	43.8–74.9	56.1	47.7	62.9
AOV_max_ (m/s)	1.32 ± 0.20	0.96–1.62	1.32	1.16	1.50
AOV_mean max_ (m/s)	0.85 ± 0.13	0.63–1.09	0.85	0.73	0.95
HR_AO_ (beats/min)	412.2 ± 85.4	240.0–600.0	420.0	360.0	480.0
EA_LV_V_max_ (m/s)	0.93 ± 0.31	0.41–1.56	0.93	0.64	1.11
HR_EALV_ (beats/min)	410.5 ± 90.4	240.0–600.0	390.0	337.5	480.0

^1^ SD: standard deviation.

**Table 6:** Doppler sonographic parameters of the examined Congo grey parrots (GPs) with left heart failure (group 2); 60 cases were created from the 46 GPs examined.

Parameter	Mean ± SD ^1^	Xmin–Xmax	Median	25% Percentile	75% Percentile
PAAT (ms)	21.2 ± 5.7	12.3–43.7	20.8	17.78	25.0
PADT (ms)	31.4 ± 7.0	17.3–55.0	26.9	27.0	36.0
PAAP (%)	40.7 ± 8.4	22.8–56.2	41.8	28.8	46.7
PADP (%)	59.8 ± 9.0	43.8–77.9	58.2	53.4	65.5
PAET (ms)	54.5 ± 9.2	37.7–92.3	54.0	49.6	58.0
PAV_max_ (m/s)	1.85 ± 0.79	0.68–4.16	1.59	1.20	2.46
PAV_mean max_ (m/s)	1.24 ± 0.53	0.50–2.45	1.07	0.82	1.53
HR_PA_ (beats/min)	425.9 ± 72.0	300.0–720.0	420.0	360.0	480.0
EA_RV_V_max_ (m/s)	0.76 ± 0.23	0.50–1.67	0.71	0.62	0.77
HR_EARV_ (beats/min)	422.7 ± 65.9	300.0–600.0	420.0	360.0	480.0
AOAT (ms)	9.77 ± 2.6	4.5–18.7	9.8	8.0	11.2
AODT (ms)	43.4 ± 9.6	25.7–63.2	42.8	35.9	50.2
AOAP (%)	18.7 ± 5.5	9.0–29.5	17.7	14.1	22.8
AODP (%)	81.1 ± 5.4	70.5–91.0	81.9	77.1	85.3
AOET (ms)	58.5 ± 10.7	39.2–99.3	57.7	50.8	66.5
AOV_max_ (m/s)	1.25 ± 0.31	0.71–2.57	1.21	1.08	1.38
AOV_mean max_ (m/s)	0.76 ± 0.18	0.41–1.49	0.73	0.64	0.86
HR_AO_ (beats/min)	419.1 ± 66.9	240.0–600.0	420.0	360.0	480.0
EA_LV_V_max_ (m/s)	0.99 ± 0.28	0.47–1.70	0.95	0.80	1.19
HR_EALV_ (beats/min)	423.7 ± 55.7	300.0–600.0	420.0	400.0	480.0

^1^ SD: standard deviation.

**Table 7:** Doppler sonographic parameters of the examined Congo grey parrots (GPs) with right heart failure or left and right heart failure (group 3); 63 cases were created from the 53 GPs examined.

Parameter	Mean ± SD ^1^	Xmin–Xmax	Median	25% Percentile	75% Percentile
PAAT (ms)	20.7 ± 6.8	7.8–38.3	18.8	16.0	26.0
PADT (ms)	40.9 ± 12.8	19.3–75.2	43.0	29.9	50.3
PAAP (%)	34.5 ± 12.0	13.1–62.2	31.8	24.7	41.3
PADP (%)	65.5 ± 12.0	37.8–86.9	68.2	58.8	75.2
PAET (ms)	62.9 ± 11.5	44.2–105.4	62.8	55.5	70.8
PAV_max_ (m/s)	1.35 ± 0.82	0.57–3.89	1.07	0.87	1.54
PAV_mean max_ (m/s)	0.90 ± 0.59	0.36–2.85	0.68	0.50	1.06
HR_PA_ (beats/min)	388.5 ± 76.1	180.0–480.0	420.0	360.0	460.0
EA_RV_V_max_ (m/s)	0.85 ± 0.30	0.28–2.14	0.80	0.65	1.01
HR_EARV_ (beats/min)	394.2 ± 82.9	150.0–540.0	420.0	360.0	480.0
AOAT (ms)	11.2 ± 3.2	5.0–19.8	10.9	9.3	12.9
AODT (ms)	46.3 ± 10.6	25.2–87.0	45.1	38.1	52.9
AOAP (%)	19.9 ± 5.5	7.9–32.6	19.4	16.3	23.3
AODP (%)	80.0 ± 5.4	67.4–92.1	80.3	76.5	82.9
AOET (ms)	62.8 ± 14.3	40.4–120.2	60.9	52.8	71.9
AOV_max_ (m/s)	1.25 ± 0.30	0.73–2.04	1.20	1.02	1.47
AOV_mean max_ (m/s)	0.76 ± 0.18	0.47–1.18	0.72	0.64	0.88
HR_AO_ (beats/min)	399.3 ± 82.3	180.0–540.0	420.0	360.0	480.0
EA_LV_V_max_ (m/s)	1.00 ± 0.28	0.41–1.90	1.00	0.80	1.15
HR_EALV_ (beats/min)	402.0 ± 75.2	180.0–540.0	420.0	360.0	480.0

^1^ SD: standard deviation.

**Table 8:** Width of the cardiac silhouette (“heart score”) measured from the ventrodorsal radiographs of the examined Congo grey parrots classified as group 1, cases without sonographic evidence of heart disease; group 2, cases with left heart failure; or group 3, cases with right or with left and right heart failure.

Heart Score (%)	Mean ± SD ^1^	Xmin–Xmax	Median	25% Percentile	75% Percentile
Group 1	49.2 ± 6.0	40.0–61.0	49.0	44.0	53.5
Group 2	52.5 ± 5.2	40.0–63.0	53.0	48.8	56.0
Group 3	56.8 ± 6.6	44.0–76.0	56.0	52.0	61.0

^1^ SD: standard deviation.

**Table 9:** Correlations of diastolic and systolic blood flow velocities and the heart rate with the shape of the blood flow profiles of the PA and AO in diseased grey parrots. The Spearman correlation coefficient (r) and the significance value (*p*) are presented. Significant correlations are highlighted in red; non-significant correlations with *p* > 0.05 and ≤0.10 are marked in green.

FP Parameter	EA_left_	EA_right_	PA	PA_mean_	AO	AO_mean_	HR
PAAT (ms)	*p* = 0.21 r = −0.08	*p* = 0.09 r = −0.12	*p* = 0.06 r = 0.12	*p* = 0.01 r = 0.16	*p* = 0.10 r = 0.11	*p* = 0.01 r = 0.16	*p* = 0.02 r = −0.21
PADT (ms)	*p* = 0.13 r = 0.10	*p* = 0.10 r = 0.11	*p* = 0.09 r = −0.11	*p* = 0.02 r = −0.15	*p* = 0.41 r = −0.05	*p* = 0.03 r = −0.14	*p* = 0.21 r = −0.12
PAET (ms)	*p* = 0.05 r = 0.13	*p* = 0.15 r = 0.10	*p* = 0.33 r = −0.06	*p* = 0.15 r = −0.09	*p* = 0.75 r = −0.02	*p* = 0.16 r = −0.09	*p* = 0.03 r = −0.20
PAAP (%)	*p* = 0.08 r = −0.12	*p* = 0.07 r = −0.12	*p* = 0.02 r = 0.15	*p* = 0.002 r = 0.20	*p* = 0.21 r = 0.08	*p* < 0.01 r = 0.18	*p* = 0.52 r = −0.06
PADP (%)	*p* = 0.09 r = 0.11	*p* = 0.09 r = 0.12	*p* = 0.02 r = −0.14	*p* = 0.004 r = −0.19	*p* = 0.23 r = −0.08	*p* < 0.01 r = 0.18	*p* = 0.46 r = −0.07
AOAT (ms)	*p* < 0.001 r = −0.36	*p* = 0.66 r = 0.04	*p* = 0.001 r = −0.30	*p* < 0.01 r = −0.32	*p* = 0.58 r = −0.05	*p* = 0.36 r = 0.08	*p* = 0.02 r = −0.26
AODT (ms)	*p* = 0.18 r = 0.12	*p* = 0.76 r = 0.03	*p* = 0.66 r = −0.04	*p* = 0.76 r = 0.03	*p* = 0.78 r = 0.03	*p* = 0.03 r = −0.18	*p* = < 0.001 r = −0.28
AOET (ms)	*p* = 0.88 r = −0.01	*p* = 0.55 r = −0.06	*p* = 0.35 r = −0.09	*p* = 0.46 r = −0.07	*p* = 0.55 r = 0.05	*p* = 0.15 r = −0.12	*p* = < 0.001 r = −0.40
AOAP (%)	*p* < 0.001 r = −0.32	*p* = 0.91 r = 0.01	*p* = 0.03 r = −0.21	*p* = 0.02 r = −0.23	*p* = 0.75 r = −0.03	*p* = 0.03 r = −0.19	*p* = 0.65 r = −0.04
AODP (%)	*p* = 0.002 r = 0.27	*p* = 0.78 r = −0.03	*p* = 0.05 r = 0.19	*p* = 0.04 r = 0.20	*p* = 0.75 r = 0.03	*p* = 0.03 r = 0.19	*p* = 0.98 r = −0.002

**Table 10:** The correlation of the heart diameter with the shape of the blood flow profile of the PA and AO in diseased Congo grey parrots. The Spearman correlation coefficient (r) and the significance value (*p*) are presented. Significant correlations are highlighted in red. Non-significant correlations of *p* > 0.05 and ≤0.1 are highlighted in green.

FP Parameter	LVd	LVs	RVd	RVs	LA	RA	FS_LV_	FS_RV_
PAAT (ms)	*p* = 0.54 r = 0.04	*p* = 0.94 r = −0.01	*p* = 0.05 r = −0.14	*p* = 0.004 r = −0.20	*p* = 0.32 r = −0.08	*p* = 0.76 r = −0.05	*p* = 0.34 r = 0.06	*p* = 0.01 r = 0.17
PADT (ms)	*p* = 0.77 r = 0.02	*p* = 0.05 r = 0.13	*p* = 0.003 r = 0.21	*p* < 0.001 r = 0.23	*p* = 0.02 r = 0.18	*p* = 0.01 r = 0.39	*p* = 0.02 r = −0.20	*p* = 0.01 r = −0.19
PAET (ms)	*p* = 0.60 r = 0.03	*p* = 0.05 r = 0.13	*p* = 0.02 r = 0.21	*p* = 0.01 r = 0.18	*p* = 0.03 r = −0.16	*p* = 0.02 r = 0.38	*p* = 0.003 r = −0.19	*p* = 0.03 r = −0.14
PAAP (%)	*p* = 0.95 r = −0.01	*p* = 0.22 r = −0.08	*p* = 0.02 r = −0.21	*p* < 0.001 r = −0.27	*p* = 0.05 r = −0.15	*p* = 0.07 r = −0.29	*p* = 0.02 r = 0.15	*p* < 0.001 r = 0.23
PADP (%)	*p* = 0.95 r = 0.01	*p* = 0.21 r = −0.08	*p* = 0.004 r = 0.20	*p* < 0.001 r = 0.26	*p* = 0.05 r = 0.15	*p* = 0.18 r = 0.21	*p* = 0.03 r = −0.15	*p* = 0.002 r = −0.21
AOAT (ms)	*p* = 0.35 r = −0.08	*p* = 0.44 r = −0.07	*p* = 0.43 r = 0.07	*p* = 0.13 r = 0.14	*p* = 0.92 r = 0.01	*p* = 0.06 r = 0.33	*p* = 0.001 r = 0.13	*p* = 0.02 r = −0.22
AODT (ms)	*p* = 0.17 r = 0.12	*p* = 0.04 r = 0.18	*p* = 0.20 r = 0.12	*p* = 0.98 r = 0.002	*p* < 0.001 r = 0.33	*p* = 0.68 r = 0.08	*p* = 0.09 r = −0.15	*p* = 0.30 r = 0.10
AOET (ms)	*p* = 0.19 r = 0.11	*p* = 0.06 r = 0.16	*p* = 0.09 r = 0.15	*p* = 0.78 r = 0.03	*p* = 0.004 r = 0.29	*p* = 0.62 r = 0.09	*p* = 0.09 r = −0.15	*p* = 0.46 r = 0.07
AOAP (%)	*p* = 0.07 r = −0.16	*p* = 0.03 r = −0.20	*p* = 0.78 r = −0.03	*p* = 0.28 r = 0.10	*p* = 0.07 r = −0.18	*p* = 0.09 r = 0.31	*p* = 0.16 r = 0.12	*p* = 0.01 r = −0.23
AODP (%)	*p* = 0.07 r = 0.16	*p* = 0.01 r = 0.23	*p* = 0.93 r = 0.01	*p* = 0.27 r = −0.10	*p* = 0.08 r = 0.18	*p* = 0.06 r = −0.34	*p* = 0.10 r = −0.15	*p* = 0.03 r = 0.20

## Data Availability

The data have been included in the text.
